# Metabolomic Analysis of Key Regulatory Metabolites in the Urine of Flavivirus-Infected Mice

**DOI:** 10.1155/2022/4663735

**Published:** 2022-06-01

**Authors:** Xiaoyan Zheng, Ran Wang

**Affiliations:** ^1^Beijing Institute of Tropical Medicine, Beijing Friendship Hospital, Capital Medical University, Beijing Key Laboratory for Research on Prevention and Treatment of Tropical Diseases, Beijing, China; ^2^Beijing Key Laboratory of Pediatric Respiratory Infection Diseases, Key Laboratory of Major Diseases in Children, Ministry of Education, National Clinical Research Center for Respiratory Diseases, Research Unit of Critical Infection in Children, Chinese Academy of Medical Sciences, 2019RU016, Laboratory of Infection and Virology, Beijing Pediatric Research Institute, Beijing Children's Hospital, National Center for Children's Health, Capital Medical University, Beijing, China

## Abstract

**Objective:**

Dengue virus (DENV), Japanese encephalitis virus (JEV), and Zika virus (ZIKV) are several important flaviviruses, and infections caused by these flaviviruses remain worldwide health problems. Different flaviviruses exhibit different biological characteristics and pathogenicity. Metabolomics is an emerging research perspective to uncover and observe the pathogenesis of certain infections.

**Methods:**

To improve the understanding of the specific metabolic changes that occur during infection with different flaviviruses, considering the principle of noninvasive sampling, this article describes our comprehensive analysis of metabolites in urine samples from the three kinds of flavivirus-infected mice using a liquid chromatography tandem mass spectrometry method to better understand their infection mechanisms.

**Results:**

The urine of DENV-, JEV-, and ZIKV-infected mice had 68, 64, and 47 different differential metabolites, respectively, compared with the urine of control mice. Among the metabolic pathways designed by these metabolites, ABC transporters, arginine and proline metabolism, and regulation of lipolysis play an important role. Furthermore, we predicted and fitted potential relationships between metabolites and pathways.

**Conclusions:**

These virus-specific altered metabolites may be associated with their unique biological properties and pathogenicity. The metabolomic analysis of urine is very important for the analysis of flavivirus infection.

## 1. Introduction

Japanese encephalitis virus (JEV), dengue virus (DENV), and Zika virus (ZIKV) are the three most common mosquito-borne flaviviruses [1], which are transmitted to humans by mosquito [2]. These flaviviruses are widely distributed in most districts of the world, causing persistent and unresolved public health problems. Importantly, although cross reactions may occur between these viruses, there are significant differences in epidemiology or clinical symptoms [3].

Urine is a complex biological sample. According to current knowledge, urine can be used as a noninvasive sample source to detect flavivirus infection. On the 14^th^ day after the onset of Japanese encephalitis (JE), live virus can be isolated from urine samples, and RNA can still be detected in urine until 26 days [4]. Similarly, the serum and urine of patients with dengue fever in the acute phase have similar positive reactions, and even if RNA is not detected in the serum, it can still be detected in the urine [5]. The viruria of ZIKV patients is well characterized [6, 7]. Studies have shown that the viruria of ZIKV may last 5 to 26 days after the onset of symptoms and lead to the excretion of infectious virus particles [8, 9], providing doctors with a potential tool to promote enhanced vector control activities in areas where flavivirus continues to spread. More importantly, urine also contains metabolic by-products excreted by the kidney, metabolites produced by intestinal flora, and all catabolites of virus-host interactions [10]. Urine-based biomarkers are particularly attractive in viral infections because urine collection is noninvasive and easy to collect, and the metabolite composition of urine is rich and comprehensive.

Metabonomics is a powerful technology that can be used to evaluate global low molecular weight metabolites (<1 kDa) in biological systems and determine the relationship between metabolic changes in biological fluids and immune system disturbances. As a mirror of the interaction between host cells and viruses, metabolomics can directly affect phenotypes rather than transcripts or proteins. Therefore, metabolomic analysis can provide obvious advantages in characterizing infection characteristics and trying to decipher the pathogenesis of disease [11]. Metabonomics attempts to capture global changes and overall physiological states in biochemical networks and pathways to clarify disturbed sites [12]. Whether there are differences in metabolomics between the above three flaviviruses on the premise that they can lead to viruria is a matter of concern. In China, it is difficult to obtain samples from JEV-infected patients due to vaccination. In addition, the local prevalence of ZIKV in China has not been reported, and scattered imported cases have been rare in recent years. We hypothesize that mice infected with JEV, DENV, or ZIKV would produce a unique characterization of the metabolic phenotype. The latest development of high-throughput molecular technology provides an opportunity to understand the pathogenesis of flavivirus infection, but the differences in urinary metabolites of several virus infections have not been clarified. Liquid chromatography tandem mass spectrometry (LC-MS/MS) has been widely used for metabolomic analysis of urine samples [13, 14]. The aim of this study was to determine the metabolic pathways associated with each flavivirus. In this article, we used LC-MS/MS metabolomic detection combined with a pattern recognition method and system analysis to perform metabolomic analysis and pathway analysis of a large biological dataset to expand our understanding of the flavivirus infection mechanism and characterize different flaviviruses and the related metabolic disturbances in urine.

## 2. Materials and Methods

### 2.1. Virus and Animals

JEV (P3 strain), DENV (serotype 2, NGC strain), and ZIKV (SMGC_1 strain) were stored at −80°C. They were used for challenge experiments. Specific pathogen-free 6-week-old female BALB/c mice were used for challenge tests.

### 2.2. Animal Experiments

Mice were randomly divided into four groups. The infected group was challenged with JEV, DENV, or ZIKV (*n* = 5 per challenge), and the control group was treated with phosphate-buffered saline (PBS) (*n* = 5). We challenged mice in the infected group with 3 × 10^5^ PFU of virus intraperitoneally.

### 2.3. Collection of Urine

Urine samples were collected 7 days after viral challenge. Generally, mice infected with flaviviruses develop acute symptoms within 1 week. Urine samples obtained by bladder massage were placed in sterile Petri dishes and stored at −80°C until use. Temporary freeze-thaw cycles are strictly avoided for urine because freeze-thaw cycles will greatly affect metabolomic test results.

### 2.4. Sample Preparation and Metabolomic Analysis

For the preparation of urine samples, we refer to a previous report [15]. LC analysis was performed on a Vanquish ultrahigh-performance liquid chromatography (UHPLC) system [15]. Relops software was used for all multivariate data analyses and model building based on principal component analysis (PCA) and partial least squares discriminant analysis (PLS-DA). Each point in the metabolic curve represents a sample. PLS-DA allows the use of variable importance on projection (VIP) in prediction to identify discriminative metabolites. *P* values, VIP, and fold change were applied to discover categorical contributing variables. Finally, metabolites with a *P*value < 0.05 and satisfying a VIP value > 1 were considered statistically significant. Pathway analysis of differential metabolites was performed by MetaboAnalyst [16, 17].

## 3. Results

We quantified the metabolite peaks in the urine of four flavivirus-infected mice. Unsupervised PCA and PLS-DA were performed on the combined metabolite profiles to visualize the clustering of samples. PCA reflects the original state of metabolome data, while PLS-DA combines regression models with dimensionality reduction and uses certain thresholds to discriminate and analyse regression results. The more aggregated the samples within a group and the more dispersed the samples among the groups, the more reliable the generated test results. Cluster analysis was used to determine the metabolic patterns of metabolites. Similar metabolic patterns strongly suggest that metabolites may have similar functions or participate in the same metabolic processes or cellular pathways. Therefore, we analysed metabolic patterns by agglomerative hierarchical clustering, clustered the same or similar metabolites, and speculated on the functions of unknown metabolites or known metabolites in mice infected with several flaviviruses to provide clinical implications. Through the analysis of VIP and *P* values in the project, 3 different metabolites of flavivirus infection and control and common differential metabolites of three groups and control were screened and identified. *P*value < 0.05 and VIP ≥1 were considered significant differential metabolites. These differential metabolites were then input into the pathway enrichment analysis.

## 4. Metabolomics Analysis of Urinary Samples from DENV-Infected Mice

**Figure 1:**
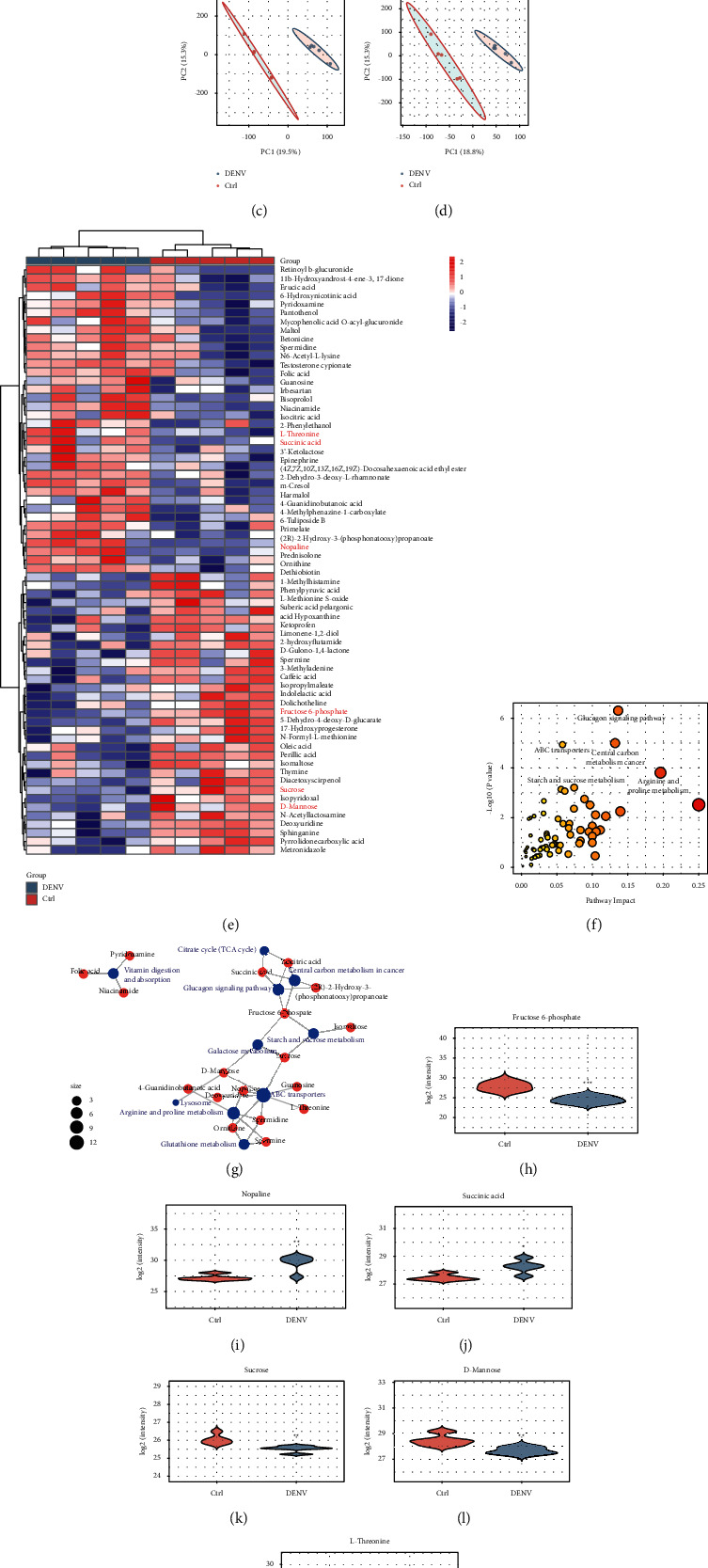
Urinary metabolite profiles reflect characteristics of DENV infections. Score plot of principal component analysis (PCA) performed using urinary metabolite data for all samples in (a) the positive-ion mode and (b) the negative-ion mode. Score plot of partial least squares (PLS) regression performed using metabolite data from samples in (c) the positive-ion mode and (d) the negative-ion mode. (e) Metabolite cluster heatmap of urine. The metabolites in urine were analysed by hierarchical clustering. Each vertical line represents a plasma sample, and rows represent metabolites. Urine samples from the same group of mice were aggregated. The relative content in the figure is displayed by different colours. The redder the colour is, the higher the expression, and the bluer the colour is, the lower the expression. The column represents the sample. (f), (g) The metabolomic view of pathway analysis using a metabolic analyser shows the most obvious metabolic pathway affected by DENV infection. (h–m) A violin diagram of metabolites was used to distinguish the DENV infection group from the control group. (h) Fructose 6-phosphate, (i) nopaline, (j) succinic acid, (k) sucrose, (l) D-mannose, and (m) L-threonine.

PCA was used to initially determine the reliability of metabolic analysis. We performed untargeted metabolomics on urine samples from mice with different flaviviruses using positive and negative-ion scanning modes to improve metabolic coverage. Although PCA showed no significant separation between control and DENV-infected mice (Figures 1(a) and 1(b)), statistical evaluation of the PLS-DA model showed that the two groups were well separated, demonstrating that urinary metabolism between DENV-infected mice and control groups showed reliable differentiation of changes (Figures 1(c) and 1(d)). Heatmap and hierarchical cluster analysis illustrated the distribution pattern of 68 different metabolites between the two groups, as shown in Figure 1(e). Potential pathways involved in differential metabolites were characterized as glucagon signalling, central carbon metabolism in cancer, ABC transporters, arginine and proline metabolism, starch and sucrose metabolism, and arginine and proline metabolism (Figures 1(f) and 1(g)). Based on the mutual confirmation of differential metabolites and potential pathways, we screened six important metabolites closely related to DENV infection, including fructose-6-phosphate, nopaline, succinic acid, sucrose, D-mannose, and L-threonine (Figures 1(h)–1(m)). Based on these results, a biologically relevant network between DENV infection and controls was formed.

### 4.1. Metabolomics Analysis of Urinary Samples from JEV-Infected Mice

**Figure 2:**
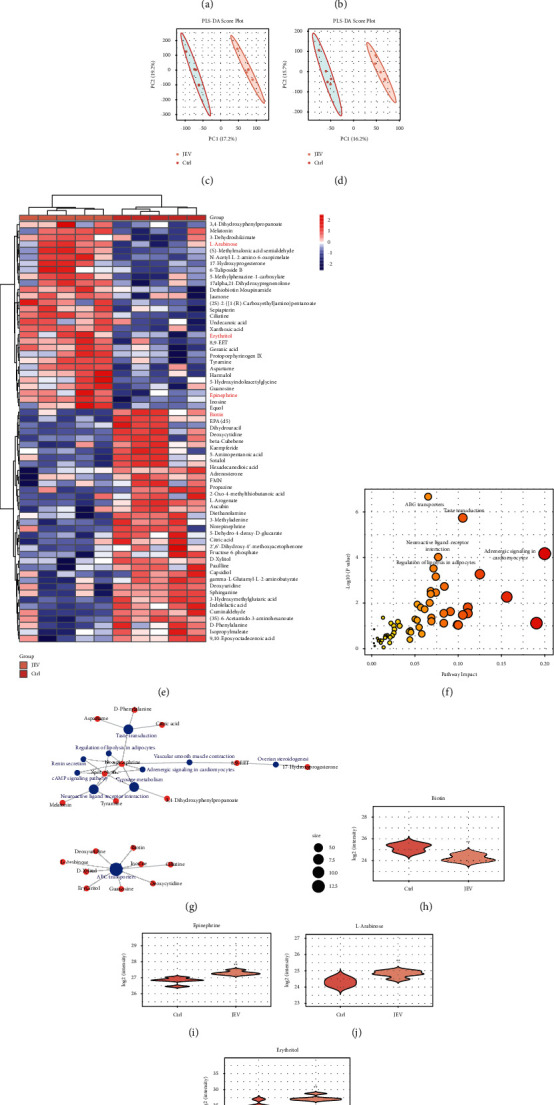
Urinary metabolite profiles reflect the characteristics of JEV infections. PCA in (a) the positive-ion mode and (b) the negative-ion mode. PLS regression in (c) the positive-ion mode and (d) the negative-ion mode. (e) Metabolite cluster heatmap of urine. (f), (g) A metabolomic view of pathway analysis to show the most obvious metabolic pathway affected by JEV infection. Violin diagram of (h–k) metabolites used to distinguish the JEV infection group from the control group. (h) Biotin, (i) epinephrine, (j) L-arabinose, and (k) erythritol.

In the first phase of data analysis, we still performed exploratory data analysis of metabolites in the urine of JEV-infected mice using PCA. A total of 37.3% and 38.0% of the total variance were explained by the first two principal components in the positive- and negative-ion modes, respectively (Figures 2(a) and 2(b)). The data show that these detected samples are arranged into two main clusters according to their groups. The sample PLS-DA results showed that the separation between the JEV-infected and control groups was more pronounced (Figures 2(c) and 2(d)). The heatmap of the found potentially differential metabolites then showed some clustering trends according to their relative intensities (Figure 2(e)). The Kyoto Encyclopedia of Genes and Genomes (KEGG) pathway was enriched using 64 common differential metabolites to generate bubble and network plots (Figures 2(f) and 2(g). The intersection of different metabolites and potential pathways was found to be the ATP-binding cassette (ABC) transporter, adrenergic signalling in cardiomyocytes, neuroactive ligand-receptor interactions, and regulation of lipolysis in adipocytes. These four pathways may play a key role in JEV infection. Biotin, epinephrine, L-arabinose, and erythritol, four important differential metabolites, occupy representative positions (Figures 2(h)–2(k)), also indicating that JEV infection and DENV infection show different metabolic changes.

### 4.2. Metabolomics Analysis of Urinary Samples from ZIKV-Infected Mice

**Figure 3:**
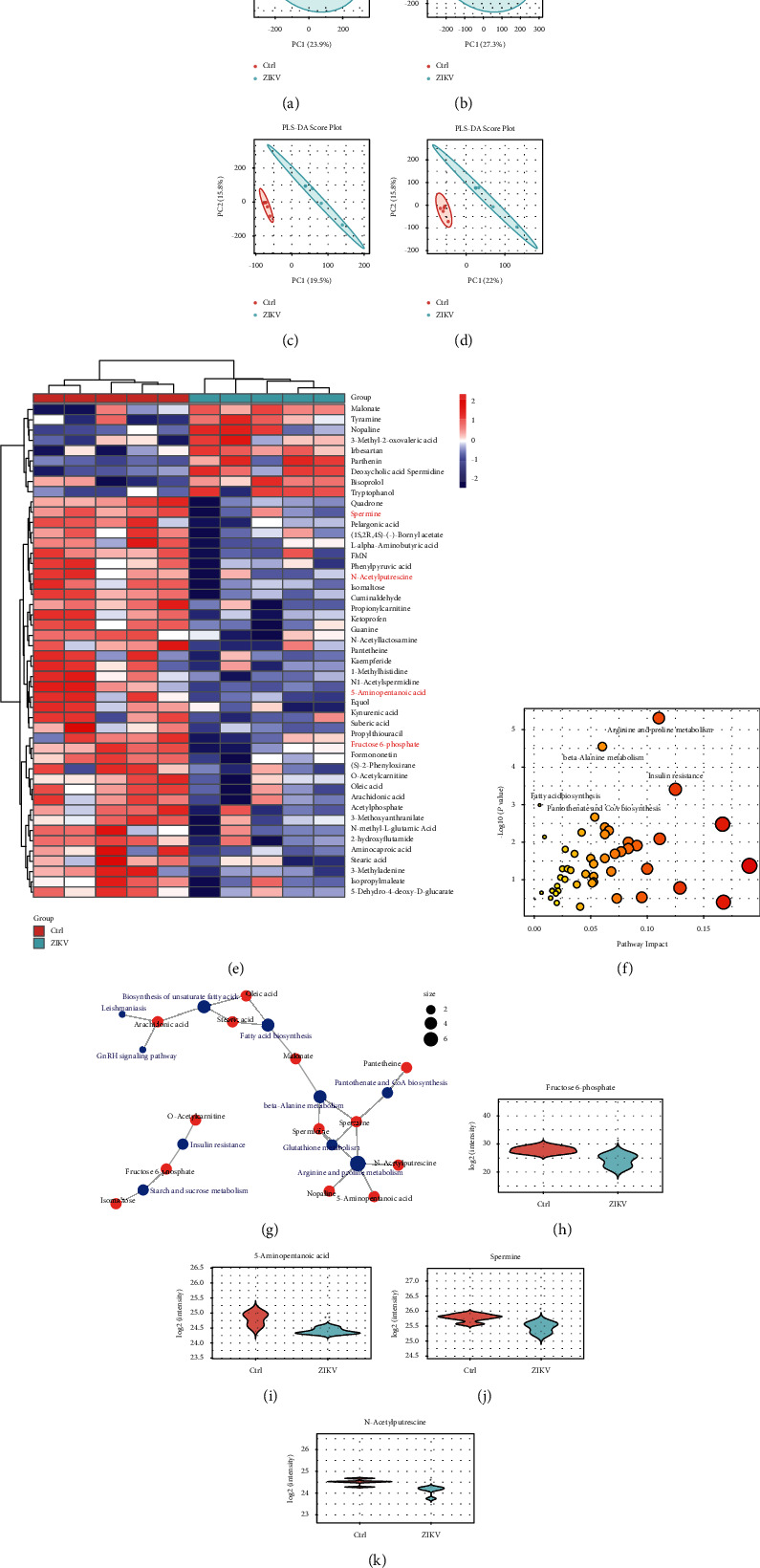
Urinary metabolite profiles reflect the characteristics of ZIKV infections. PCA in (a) the positive-ion mode and (b) the negative-ion mode. PLS regression in (c) the positive-ion mode and (d) the negative-ion mode. (e) Metabolite cluster heatmap of urine. (f), (g) A metabolomic view of pathway analysis to show the most obvious metabolic pathways affected by ZIKV infection. (h–k) Violin plot of metabolites used to differentiate ZIKV infection from controls. (h) Fructose 6-phosphate, (i) 5-aminopentanoic acid, (j) spermine, and (k) N-acetylputrescine.

Multivariate statistical analysis revealed that in the PCA model, the metabolic profiles of ZIKV-infected and control groups were indistinguishable and largely overlapped, which was very different from DENV and JEV infections (Figures 3(a) and 3(b)). However, in the PLS-DA model, the two groups showed significant differences (Figures 3(c) and 3(d)). We generated a heatmap of differential metabolites to more clearly represent the differences between groups of metabolites in urine, and the results are shown in Figure 3(e). After database search and screening, a total of 47 differential metabolites were identified in urine samples, which is less than the number of differential metabolites in JEV and DENV infections. Next, we identified the 4 metabolic pathways most associated with ZIKV infection, namely arginine and proline metabolism, *β*-alanine metabolism, insulin resistance, and pantothenic acid and CoA biosynthesis (Figures 3(f) and 3(g)). Among these pathways, the arginine and proline metabolic pathways are shared with those involved in DENV infection. We combined differential metabolites with related metabolic pathways and found that fructose-6-phosphate, 5-aminovaleric acid, spermine, and N-acetylputrescine may be involved in these metabolic pathways (Figures 3(h)–3(k)).

### 4.3. Multigroup Metabolomics Analysis of Urinary Samples from Flavivirus-Infected Mice

**Figure 4:**
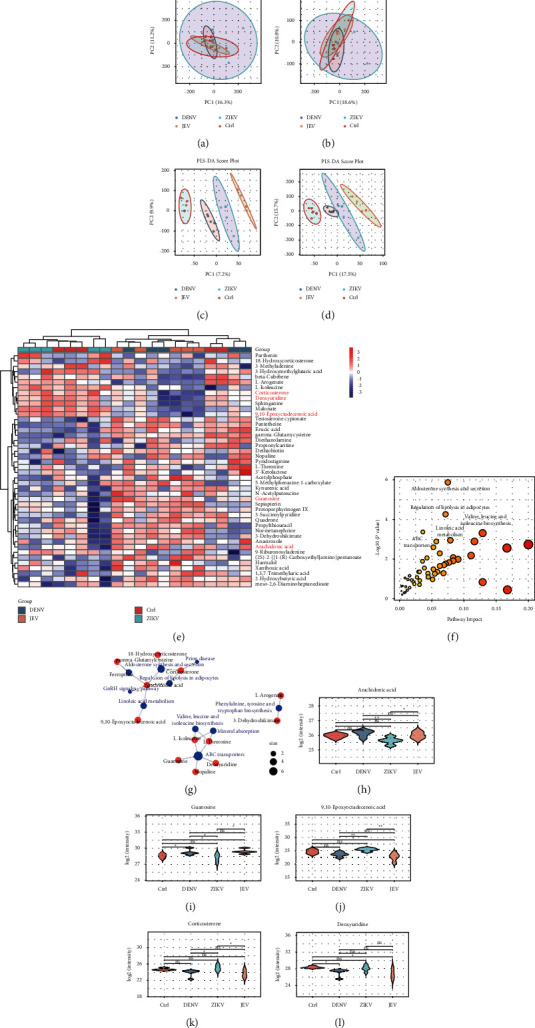
Urinary metabolite profiles reflect the characteristics of flavivirus infections. PCA in (a) the positive-ion mode and (b) the negative-ion mode. PLS regression in (c) the positive-ion mode and (d) the negative-ion mode. (e) Metabolite cluster heatmap of urine. (f), (g) Metabolomic view of pathway analysis to show the most obvious metabolic pathways affected by flavivirus infection. (h–l) Violin plots of metabolites used to differentiate ZIKV infection from controls. (h) Arachidonic acid, (i) guanosine, (j) 9,10-epoxyoctadecenoic acid, (k) corticosterone, and (l) corticosterone.

The PCA plots are shown in Figures 4(a) and 4(b), and each group of samples is not well differentiated. Likewise, we examined the PLS score plot and confirmed that the model successfully identified metabolite trends corresponding to viral infection, with the control and JEV samples falling on the opposite ends of the plot, while the DENV and ZIKV samples were in the middle (Figures 4(c) and 4(d)), which suggests different urine metabolic profiles due to different flaviviruses. As shown in Figure 4(e), further heatmap analysis of these compounds revealed several clusters. Through pathway enrichment and pathway topology analysis of flavivirus-infected mice, four important metabolic pathways were identified: aldosterone synthesis and secretion, regulation of lipolysis in adipocytes, ABC transporters, and linoleic acid metabolism (Figures 4(f) and 4(g)). We speculate that two of the four potential pathways are identical to those in DENV and JEV infections but different from the pathways in ZIKV infection, suggesting a unique metabolic pattern following ZIKV infection detected in urine. The 4 representative differential metabolites did not overlap with the representative differential metabolites for DENV, JEV, and ZIKV infections, reflecting differences in commonality and personality (Figures 4(h)–4(l)).

## 5. Discussion

Metabolomics may play an important role in the study of infectious disease metabolites [18]. Urine metabolomic analysis can provide a more comprehensive understanding of metabolite alterations in flavivirus infection [19]. In this study, an untargeted LC-MS/MS metabolomic analysis platform was used to analyse the changes in metabolite levels in the urine of DENV, JEV, ZIKV, and uninfected control mice. To our knowledge, this is the first study to measure the metabolomics of flavivirus infection using urine samples. The results showed that several common flavivirus infections caused metabolic levels different from the levels in the control group, and subsequent metabolic pathway analysis revealed that several virus-altered metabolites were involved mainly in different biochemical pathways. Symptomatic flavivirus infections are mostly acute in course, and there has been great interest in determining whether metabolomic analysis can identify individuals with clinically symptomatic flavivirus infections. By design, in this study, urine metabolite analysis of flavivirus-infected mice showed differences from controls. Symptomatic flavivirus infections are mostly acute in the course of disease, and there is great interest in determining whether metabolomic analysis can identify individuals with clinically symptomatic flavivirus infections. By design, in this study, urine metabolite analysis of flavivirus-infected mice showed differences from controls.

In DENV infection, glucagon signalling, central carbon metabolism, ABC transporters, arginine and proline metabolism, and starch and sucrose were altered. Notably, the role of glucagon signalling and starch and sucrose metabolism in infection has not been reported. Alterations in central carbon metabolism and arginine and proline metabolism have only been reported in fungal, parasitic, and bacterial infections [20–22]. Importantly, ABC transporters represent a heterogeneous group of ATP-dependent transporters widely involved in various physiological processes in various tissues. ABC transporters also regulate the development and function of CD8^+^*T* and CD4^+^ T cells, affecting immunity to viruses [23]. In this pathway, we found that L-threonine and nopaline were upregulated and D-mannose was downregulated, suggesting that these metabolites play a role in the DENV infection mechanism. JEV infection also interferes with this pathway, suggesting that the pathophysiological processes of DENV and JEV infection are similar.

For JEV infection, biotin was downregulated and L-arabinose and erythritol were upregulated in the ABC transporter pathway, suggesting that ABC transporter pathway-mediated T-cell immunity is crucial in JEV infection-mediated pathogenesis, which is closely related to JEV infection-mediated pathogenesis. The results for DENV infection mentioned earlier were similar. In addition to changes in ABC transporters, adrenergic signalling, neuroactive ligand-receptor interactions, and regulation of lipolysis may also be involved in the pathogenic mechanism of JEV infection. Adrenergic signalling is required to control the adaptive response of natural killer (NK) cells [24]. Upregulated epinephrine may be a protective factor against JEV infection. At the same time, epinephrine is also a common node for neuroactive ligand-receptor interactions and adrenergic signalling and regulation of lipolysis. Lin et al. reported that influenza virus infection can significantly upregulate the expression of genes related to the neuroactive ligand-receptor interaction pathway, thereby participating in synaptic transmission [25]. We speculated that this pathway may be related to central nervous system disease induced mainly by JEV infection. In particular, this result suggests that targeting the neuroactive ligand-receptor interaction pathway may provide a strategy to control JEV-induced encephalopathy and encephalitis. By contrast, the role of lipolytic regulation in JEV infection is not well understood.

Compared with DENV and JEV, ZIKV caused fewer changes in metabolite species, probably because BALB/c mice did not develop obvious symptoms because this strain of mice is not a susceptible model of ZIKV. Pang et al. have previously reported extensive and large-scale metabolic reprogramming events in the brains of ZIKV-infected mice [26]. Here, we found different characteristics of urine samples. Downregulated fructose-6-phosphate suggests that ZIKV infection may affect pathogenesis by interfering with the insulin-resistance pathway. Potential regimens for insulin resistance have received attention in the treatment of hepatitis C virus and human immunodeficiency virus [27,28]. Spermine downregulation affects pantothenic acid and CoA biosynthesis. Pantothenic acid is an important precursor for the synthesis of the cofactor CoA. In fact, pantothenic acid and CoA biosynthesis have been shown to be promising antiparasitic drug targets [29]. The role of the *β*-alanine metabolic pathway in ZIKV infection remains to be studied.

From the metabolomic differences between the three flaviviruses and the control group, the prominent locations of ABC transporters and the regulation of lipolysis reappeared, suggesting that the metabolic changes caused by these flaviviruses have commonalities. However, the specific metabolites involved in this process differ, mainly arachidonic acid and corticosterone, which also play a role in the aldosterone synthesis and secretion pathway. The effect of arachidonic acid is also related to the metabolism of linoleic acid. The roles of arachidonic acid and corticosterone, mainly in ZIKV infection, are particularly pronounced. Saini et al. demonstrated the prophylactic and therapeutic importance of linoleic acid in the control of Leishmania infection [30]. Further studies are required to determine the mechanistic significance of these altered metabolites in flavivirus infection.

The limitations of our study should be noted. Our conclusions are drawn from experiments in mice. Urine samples from infected mice were examined by untargeted metabolomics. Therefore, the results and conclusions for these metabolites should be interpreted with caution, and our findings should be validated for clinical utility in clinical patients.

## 6. Conclusions

In conclusion, the present study provides relatively broad metabolite coverage in mouse assays following flavivirus infection by using a nontargeted LC-MS/MS approach for urine metabolomic analysis, allowing the discovery of up-/downregulated expression of novel metabolites. These apparent differences may reflect their onset and progression in flavivirus infections and may provide important references for new clinical applications. However, these results are exploratory, and further external validation studies are needed to support these observations and clarify their role in flavivirus infection-specific metabolic alterations.

## Data Availability

The data used to support the findings are included within the article.
